# Characterization of Two Homogalacturonan Pectins with Immunomodulatory Activity from Green Tea

**DOI:** 10.3390/ijms15069963

**Published:** 2014-06-04

**Authors:** Huijun Wang, Guodong Wei, Fei Liu, Gautam Banerjee, Manoj Joshi, S. W. Annie Bligh, Songshan Shi, Hui Lian, Hongwei Fan, Xuelan Gu, Shunchun Wang

**Affiliations:** 1 The MOE Key Laboratory for Standardization of Chinese Medicines and the SATCM Key Laboratory for New Resources and Quality Evaluation of Chinese Medicines, Institute of Chinese Materia Medica, Shanghai University of Traditional Chinese Medicine, 1200 Cailun Road, Shanghai 201203, China; E-Mails: wanghuijun666666@163.com (H.W.); feilion_01@hotmail.com (F.L.); shisongshan1978@126.com (S.S.); 15297626906@163.com (H.L.); fhwei_love@126.com (H.F.); 2 Unilever R&D Shanghai, 66 Lin Xin Road, Linkong Economic Development Zone, Shanghai 200335, China; E-Mail: wgd992003@126.com; 3 Unilever R&D Bangalore, 66 Main Road, Whitefield, Bangalore 560066, India; E-Mails: Gautam.Banerjee@unilever.com (G.B.); Joshi.manoj@unilever.com (M.J.); 4 Department of Complementary Medicine, Faculty of Science and Technology, University of Westminster, London W1W 6UW, UK; E-Mail: a.bligh@westminster.ac.uk

**Keywords:** *Camellia sinensis*, green tea, homogalacturonan, pectin, phagocytosis, immunomodulatory

## Abstract

Two natural homogalacturonan (HG) pectins (*M*_W_ *ca.* 20 kDa) were isolated from green tea based on their immunomodulatory activity. The crude tea polysaccharides (TPS1 and TPS2) were obtained from green tea leaves by hot water extraction and followed by 40% and 70% ethanol precipitation, respectively. Two homogenous water soluble polysaccharides (TPS1-2a and TPS1-2b) were obtained from TPS1 after purification with gel permeation, which gave a higher phagocytic effect than TPS2. A combination of composition, methylation and configuration analyses, as well as NMR (nuclear magnetic resonance) spectroscopy revealed that TPS1-2a and TPS1-2b were homogalacturonan (HG) pectins consisting of a backbone of 1,4-linked α-d-galacturonic acid (GalA) residues with 28.4% and 26.1% of carboxyl groups as methyl ester, respectively. The immunological assay results demonstrated that TPS1-2, which consisted mainly of HG pectins, showed phagocytosis-enhancing activity in HL-60 cells.

## 1. Introduction

Phagocytosis plays an important role in the defence of humans and animals from infectious and non-infectious agents. In the immune system, macrophages act as regulatory and effector cells. Therefore enhancement of phagocytic function by therapeutic intervention will be beneficial in treatments of microbial infection, cancer, inflammation, and ageing [1,2].

Pectin is structurally and functionally the most complex polysaccharide in plant cell walls. Pectin is a family of galacturonic acid-rich polysaccharides including homogalacturonan (HG), rhamnogalacturonan I (RG-I), and the substituted galacturonans, such as rhamnogalacturonan II (RG-II), and xylogalacturonan (XGA) [3]. Xu *et al.* [4] has isolated and characterized a homogalacturonan from the radix of *Platycodon grandiflorum*. RG-I and RG-II pectins have been obtained from green tea leaves by Ele-Ekouna *et al.* [5]. Plant cell wall polysaccharides (such as pectins) are well known to possess a diverse immunomodulating activity that can mediate both phagocytosis and antibody production [1]. The wide structural diversity of plant cell wall polysaccharides reflects the different mechanisms exerted on the immune systems. These polysaccharides can be used to stop, prevent or heal various infections or pathology. Some of their sub-fractions are also able to activate the animal immune system [6].

Tea, collected from the plant *Camellia sinensis* L., is one of the most widely consumed beverages in the world. Green tea is increasingly recognized as a health drink because many of its bioactive components have been characterized and studied [7], such as polyphenols [8,9,10], polysaccharides [5,11], alkaloids [12], amino acids [13], and nucleic acids [14]. Tea polysaccharides (TPS), which exist as a structural constituent of the cell walls of tea plants [15], have shown immune enhancement [16], blood sugar lowering [17,18,19] and anti-cancer activities [20,21]. However, the structures of some active polysaccharides of green tea have not been well studied. For example, previous studies reported that tea polysaccharides having an immunomodulatory effect were mainly composed of uronic acids, but their structures were not characterised [22].

In this paper, we isolated two homogeneous acidic polysaccharides from green tea with immunomodulatory activity. Their chemical structures were identified using a combination of composition, periodate oxidation, methylation and configuration analyses, as well as 1D and 2D NMR spectroscopy.

## 2. Results and Discussion

### 2.1. Isolation of TPS1-2a and TPS1-2b Based on Immunomodulatory Activity

**Figure 1:**
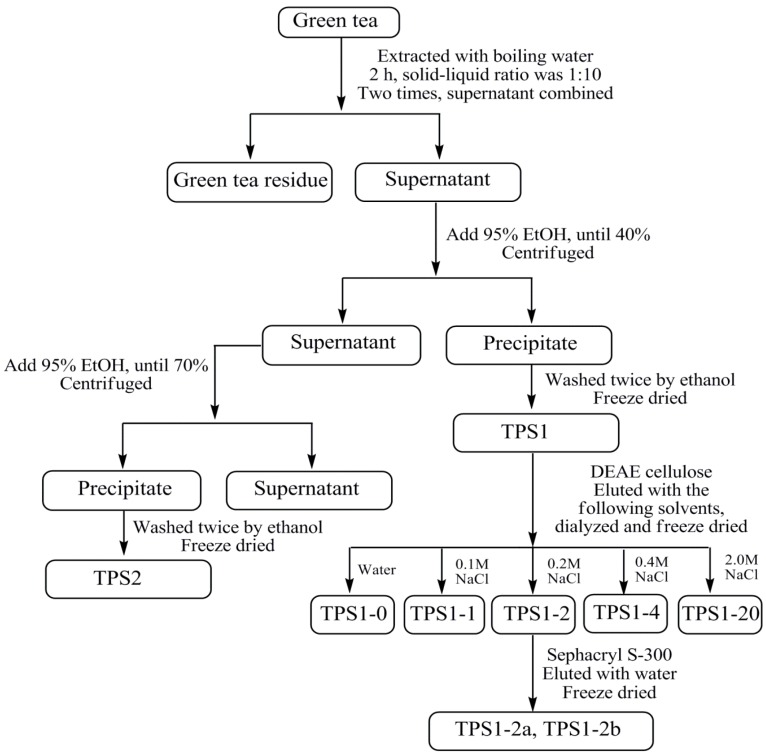
Flow chart of the isolation of aqueous polysaccharides from green tea.

**Figure 2:**
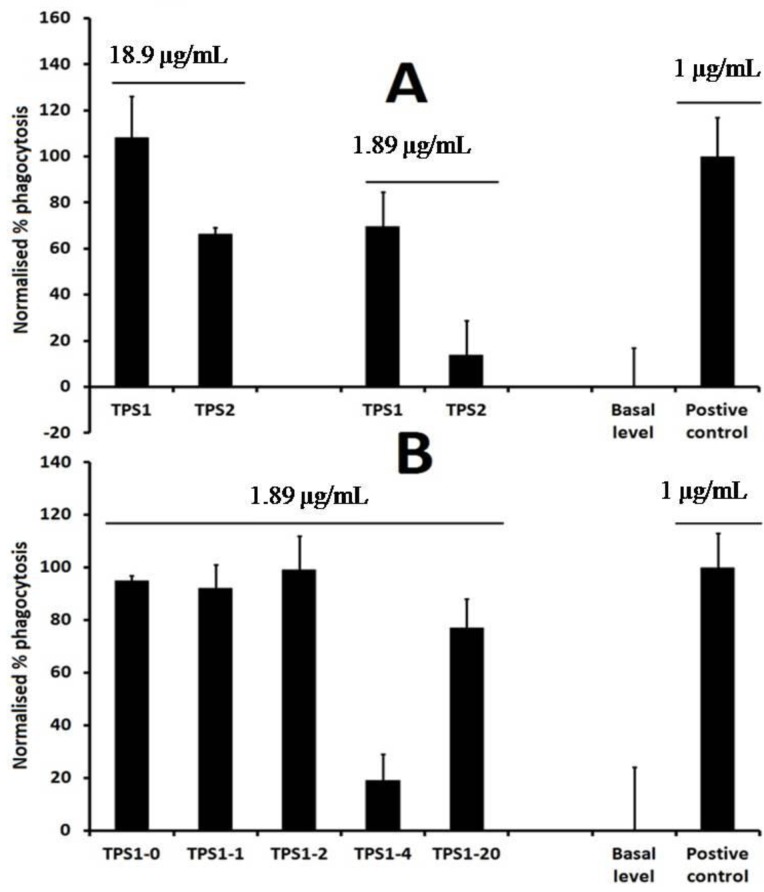
Phagocytic activity of (**A**) TPS1, TPS2 (18.9 or 1.89 μg/mL) and (**B**) sub-fractions of TPS1 (1.89 μg/mL). Positive control (LPS, 1 μg/mL).

**Figure 3:**
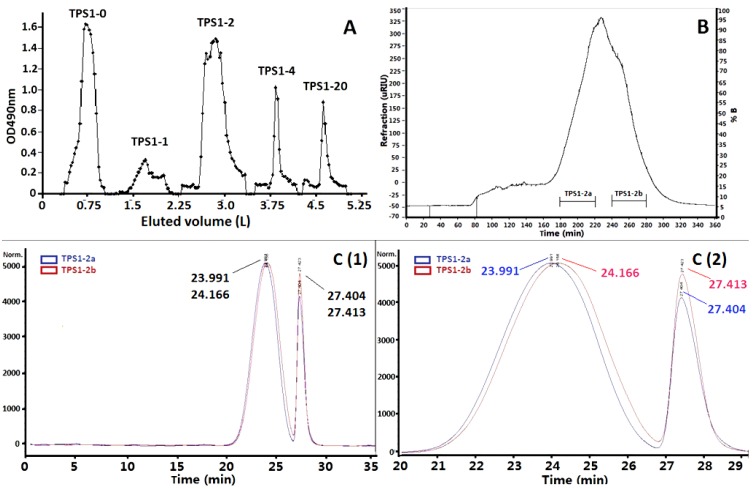
(**A**) Profile of TPS1 in DEAE-cellulose column (Tube volume: 15 mL); (**B**) TPS1-2 in High-Resolution Sephacryl™ S-300 (TPS1-2a: 180–220 min and TPS1-2b: 240–280 min), and TPS1-2a and TPS1-2b in HPGPC (**C** (**1**), 0–35 min; **C** (**2**), 20–29 min, a small gap at *Y*-axis of NaCl peaks as the eluants of slightly different NaCl concentration were used to elute).

The crude tea polysaccharides (TPS1 and TPS2) were obtained from green tea leaves by hot water extraction and followed by 40% and 70% ethanol precipitation, respectively (Figure 1). Phagocytosis assay results showed TPS1 was more potent than TPS2 at the concentration of 1.89 and 18.9 μg/mL, and TPS2 did not show any phagocytosis-enhancing activity at the concentration of 1.89 μg/mL (Figure 2A). TPS1 was further fractionated on a DEAE (dicthylaminoethyl)-cellulose column, which was eluted stepwise with distilled water, 0.1, 0.2, 0.4 and 2.0 M NaCl solutions to give TPS1-0 (water fraction, yield 1.12%), TPS1-1 (0.1 M NaCl fraction, yield 1.82%), TPS1-2 (0.2 M NaCl fraction, yield 12.86%), TPS1-4 (0.4 M NaCl fraction, yield 4.46%) and TPS1-20 (2.0 M NaCl fraction, yield 3.88%) (Figure 3A). The sugar content was detected by the phenol-sulfuric acid method. The normalised % phagocytosis of the sub-fractions of TPS1 are shown in Figure 2B. The results showed TPS1-0 TPS1-1 and TPS1-2 fractions have the same phagocytic activity, but are higher than other sub-fractions. Considering both the good activity and yield, TPS1-2 was further fractionated using Sephacryl™ S-300 high resolution column (Figure 3B), to obtain carbohydrate fractions of TPS1-2a (yield 20.0% from TPS1-2) and TPS1-2b (yield 22.5% from TPS1-2). Both TPS1-2a and TPS1-2b were homogeneous, as they were eluted at a single symmetrical peak (the second peak was the NaCl peak) from high performance gel permeation chromatography (HPGPC) as shown in Figure 3C, (1) and (2), with a molecular weight of 22 and 20 kDa, respectively. 

### 2.2. Monosaccharide Analysis and Degree of Esterification

Complete hydrolysis of TPS1-2a and TPS1-2b followed by TLC (thin layer chromatography) showed that both polysaccharides contained only uronic acid. This was confirmed by the *m*-hydroxybiphenyl method [23]. Both TPS1-2a and TPS1-2b were found to contain 100% of uronic acid using d-galacturonic acid as a standard.

**Figure 4:**
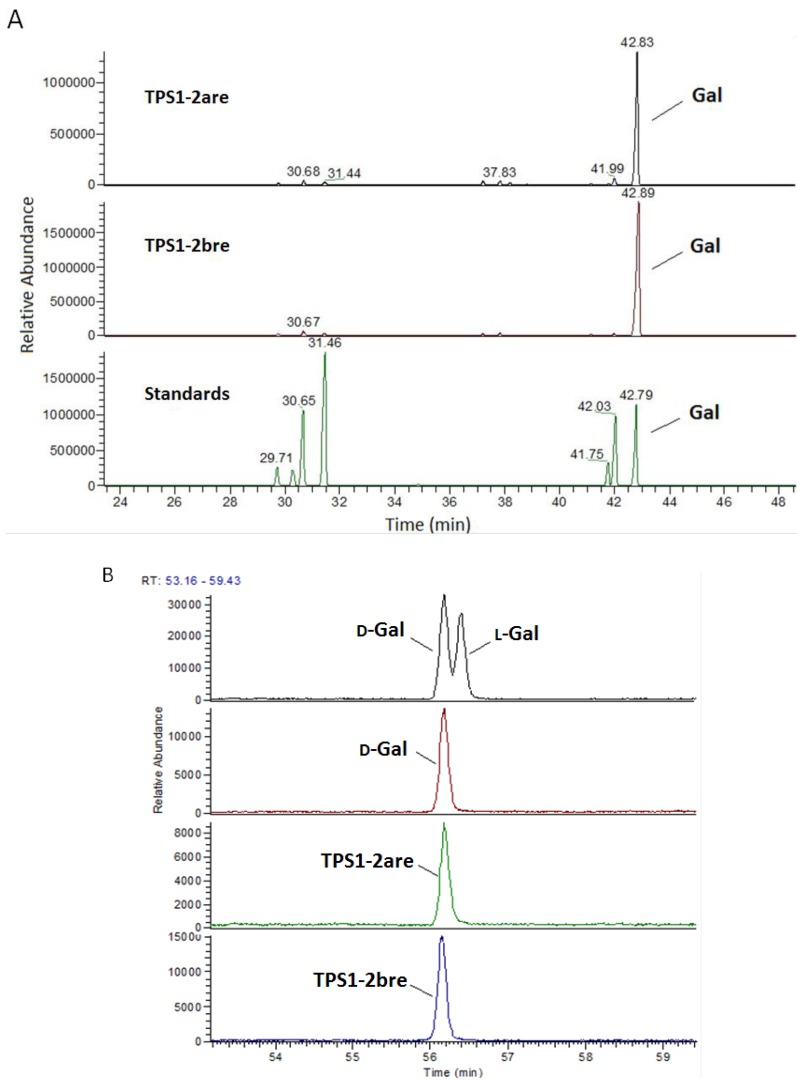
(**A**) Monosaccharide composition of TPS1-2are and TPS1-2bre, reduced form of TPS1-2a and TPS1-2b, respectively; (**B**) Configuration of TPS1-2are and TPS1-2bre comparing to d-Gal and l-Gal standards.

TPS1-2a and TPS1-2b were reduced three times with sodium borohydride by Taylor’s method [24] to give the carboxyl-reduced polysaccharides, TPS1-2are and TPS1-2bre. Complete hydrolysis of TPS1-2are and TPS1-2bre followed by TLC analysis also indicated that they did not contain uronic acid. After the hydrolysates were converted into their corresponding alditol acetates and analyzed by GC-MS, both TPS1-2are and TPS1-2bre, only galactose resulted from galacturonosyl residues present in TPS1-2a and TPS1-2b was found (Figure 4A). The configuration of galactose in TPS1-2are and TPS1-2bre (Figure 4B) were assigned as the D configuration by comparing them with d-galactose and l-galactose standards using the GC-MS method developed by Cases *et al.* [25]. Hence d-galacturonic acid is the major uronic acid present in TPS1-2a and TPS1-2b with about 28.4% and 26.1% of carboxylic groups present as methyl ester in galacturonic acid residues, respectively. The degrees of *O*-acetylation at 2-*O* and/or 3-*O* were determined as only 0.48% and 0.79% for TPS1-2a and TPS1-2b.

### 2.3. Periodate Oxidation

According to the results of periodate oxidation, 1 mol of GalA residues in TPS1-2a and TPS1-2b consumed 0.97 and 0.98 mol NaIO_4_, respectively, in approximate accordance with a calculated value of 1, suggesting the linkage of TPS1-2a and TPS1-2b was either 1→4 or 1→2. 

### 2.4. Methylation Analysis

TPS1-2are and TPS1-2bre were methylated three times to give completely methylated polysaccharides. GC-MS analysis indicated that both TPS1-2are and TPS1-2bre were linear (1,4)-linked galactans (Table 1), inferring that the native TPS1-2a and TPS1-2b were linear (1,4)-linked galacturonic acid residues.

**Table 1:** GC-MS data for methylation analysis of TPS1-2are and TPS1-2bre, reduced form of TPS1-2a and TPS1-2b, respectively.

Methylation Sugars	Linkages	Molar Ratio (%)		Major Mass Fragments (*m*/*z*)
		TPS1-2are	TPS1-2bre	
2,3,6-Me_3_-Gal*p*	1,4-Gal*p*	89.28	87.84	45,87,99,101,113,117,129,131,143,161,173,233
2,3,4,6-Me_4_-Gal*p*	Terminal	10.72	12.16	45,71,87,101,117,129,145,161,205

### 2.5. NMR (Nuclear Magnetic Resonance) Spectroscopy

**Figure 5:**
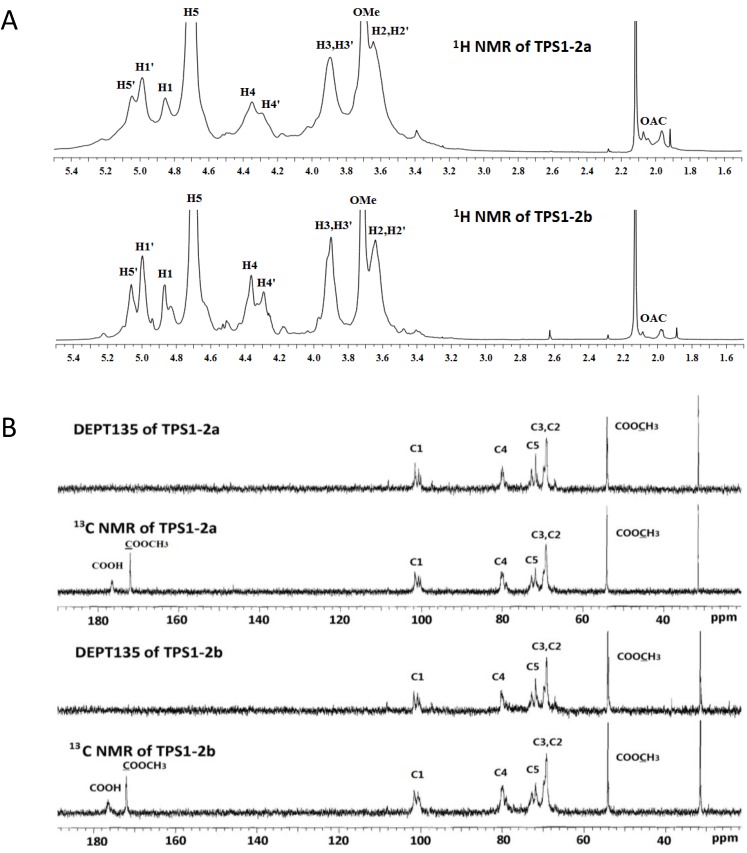
(**A**) ^1^H NMR (nuclear magnetic resonance); (**B**) ^13^C and DEPT135 NMR spectra of TPS1-2a and TPS1-2b.

The ^13^C and DEPT135 NMR spectra of TPS1-2a and TPS1-2b (Figure 5) were assigned, according to the literature values [26,27]. The signal at 176.5 ppm, which did not exist at DEPT135 NMR, was assigned to C-6 of (1,4)-linked GalA. The signal at 172.0 ppm, also disappeared at DEPT135 NMR, and that at 54.1 ppm suggested that partial GalA residue might exist as a methyl ester. In the anomeric carbon region, the signals at 100.7–101.7 ppm indicated an α-anomeric configuration in galacturonic acid residue units. The signal at the region 79.0 and 79.8 ppm was assigned to methyl and nonmethyl esterified C-4, and those at 69.2–69.8 ppm to C-2 and C-3. The signal at 71.8 and 72.7 ppm attributed to nonmethyl and methyl esterified C-5 of (1,4)-linked GalA. The methylene signals of DEPT135 NMR, which should appear as negative peaks, could not be detected, inferring that no neutral sugars were present in TPS1-2a and TPS1-2b (Figure 5). 

**Figure 6:**
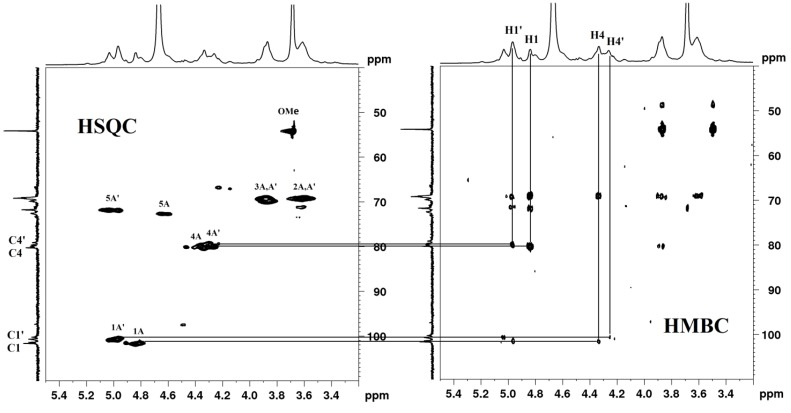
HSQC (heteronuclear singular quantum correlation) and HMBC (heteronuclear multiple bond correlation) spectra of TPS1-2a and/or TPS1-2b.

The ^1^H NMR signals of TPS1-2a and TPS1-2b were assigned by HSQC (heteronuclear singular quantum correlation), HMBC (heteronuclear multiple bond correlation) (Figure 6) and literature data [4,28]. The signal at 3.71 ppm showed the characteristics of protons of methyl ester existed in (1,4)-linked GalA. The signals at 4.87 and 5.00 ppm were assigned to nonmethyl and methyl esterified H-1 of (1,4)-linked GalA, which also indicated that the GalA residues possessed an α configuration [4]. The signals at 3.64 and 3.90 ppm were assigned to H-2 and H-3 of (1,4)-linked GalA. The signals at 4.70 and 5.05 ppm were attributed to nonmethyl and methyl esterified H-5 of (1,4)-linked GalA. HMBC spectrum of TPS1-2a and TPS1-2b showed clear correlations between H-1 and C-4 of nonmethyl and methyl esterified (1,4)-linked GalA; and between H-4 and C-1 of nonmethyl and methyl esterified (1,4)-linked GalA (Figure 6). The ^13^C and ^1^H chemical shifts assignments were shown in Table 2.

**Table 2:** ^13^C NMR and ^1^H NMR chemical shifts (ppm) for TPS1-2a and/or TPS1-2b.

Residues	C-1/H-1	C-2/H-2	C-3/H-3	C-4/H-4	C-5/H-5	C-6/H-6	OCH_3_
→4)-α-GalA-(-1→	101.7	69.2	69.8	79.8	72.7	176.5	-
(TPS1-2a/2b, A) *	4.87	3.64	3.90	4.36	4.70	-	-
→4)-α-GalA6Me-(-1→	100.7	69.2	69.8	79.0	71.8	172.0	54.1
(TPS1-2a/2b, A') *	5.00	3.64	3.90	4.29	5.06	-	3.71

* (A): Nonmethyl esterified (1,4)-linked GalA; (A'): Methyl esterified (1,4)-linked GalA.

### 2.6. Summary on the Structure of TPS1-2a and TPS1-2b

**Figure 7:**
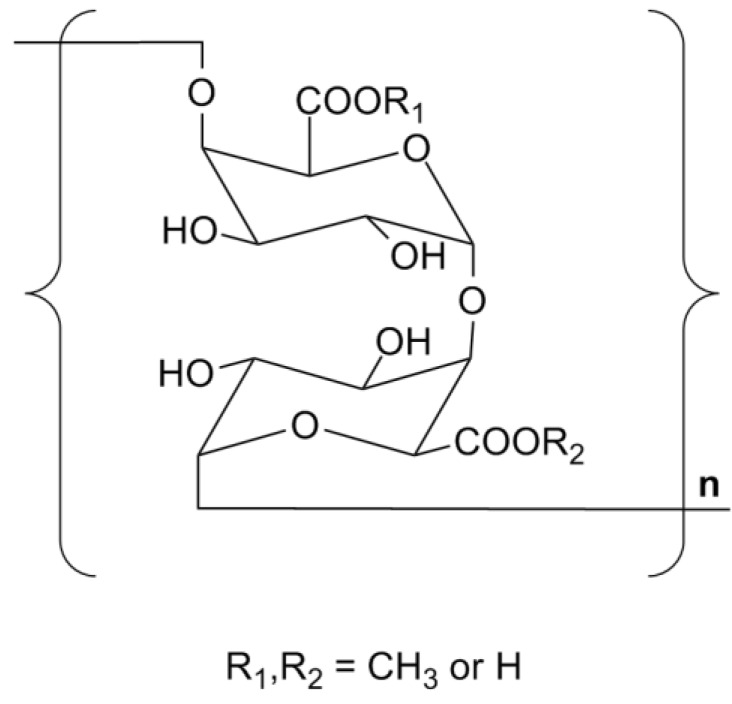
The basic chemical unit of TPS1-2a and TPS1-2b.

Based on the results, it can be concluded that both TPS1-2a and TPS1-2b are linear (1,4)-α-d-galacturonan with molecular weights of 22 and 20 kDa, and their degrees of methyl-esterification are 28.4% and 26.1%, while the degrees of substitution of *O*-acetylation were 0.48% and 0.79%, respectively. They can be classified as a typical homogalacturonan (HG) polymer [29] and their monomeric unit is shown in Figure 7.

### 2.7. Discussion

In the present study, two homogalacturonan (HG) pectins (*M*_W_ *ca.* 20 kDa) were isolated from green tea based on their immunomodulatory activity. The chemical and spectral characterization indicated the two HG pectins (TPS1-2a and TPS1-2b) are linear (1,4)-α-d-galacturonan with 28.4% and 26.1% of carboxyl groups as methyl ester, respectively, which are similar to the polysaccharides we have isolated from green tea residue provided by polyphenols manufacturing (Shanghai Novanat Bioresources Co., Ltd., Shanghai, China) [23]. The basic chemical structure of these homogeneous polysaccharides is shown in Figure 7. 

HG polymer is a major type of pectin found in many different plant sources, and its composition varied greatly among different sources [30,31,32,33,34]. Previous studies reported that HG pectin was able to stimulate M1-polarized macrophages and promote Th1-oriented adaptive immune response even at a low concentration (20 μg/mL). It also induced TNF-α secretion by human peripheral blood mononuclear cells, and reduced arginase activity, but did not affect IL-10 secretion by murine macrophages or human peripheral blood mononuclear cells. The IL-12 and NO-stimulating effects on murine macrophages were similar to that of LPS [35]. In this study we have shown that the TPS1-2 sub-fraction at 1.89 μg/mL gave similar phagocytosis effect as LPS (1 μg/mL). 

Previous studies also reported that tea polysaccharides have an immunomodulatory effect *in vivo* and *in vitro* [36,37]. Wang *et al.* [37] reported that tea polysaccharides had the potential of promoting phagocytic activity of monocyte-macrophage system in rats. CS-F2, an acidic polysaccharide was isolated from green tea but its structure was not characterised fully. In this work we showed that an acidic polysaccharide TPS1-2 consisted mainly of HG pectin and exhibited high phagocytosis-enhancing activity in HL-60 cells. This connection between HG pectin and immunomodulatory effect highlights the significance of these polysaccharides from green tea in promoting immune enhancement. 

## 3. Experimental Section

### 3.1. Materials and Reagents

The commercial sample of Wufeng green tea leaves was purchased in June, 2010 from a market in Wuhan, Hubei province of China. DEAE-cellulose column and High-Resolution Sephacryl™ S-300 were obtained from GE Healthcare Bio-Sciences AB (Uppsala, Sweden). Monosaccharide standards (d-glucose, d-galactose, d-arabinose, l-rhamnose, d-manonose, d-xylose, d-galacturonic acid) and 1-cyclohexyl-3-(2-morpholinoethyl) carbodiimidemetho-*p*-toluenesulphonate (CMC) were purchased from Sigma-Aldrich (St. Louis, MO, USA). All the reagents used were of analytical grade.

### 3.2. General Methods

High performance gel permeation chromatography (HPGPC) was performed with an Agilent 1100 instrument (Agilent, Santa Clara, CA, USA) fitted with the GPC software, using KS-805 and KS-804 connection in series (Shodex Co., Tokyo, Japan). Gas chromatography (GC) was performed with a TRACE GC apparatus (Thermo Fisher Scientific, Waltham, MA, USA) equipped with a DB-624 column (length: 30 m × 0.32 mm, thickness of liquid phase: 1.8 μm, Agilent) for the determination of *O*-methyl group. GC-MS was analyzed with a TRACE DSQ apparatus (Thermo Fisher Scientific) equipped with a TR-5 column (length: 60 m × 0.25 mm, thickness of liquid phase: 0.25 μm, Thermo Fisher Scientific). The GC-MS temperature program used for monosaccharide analysis was 140–198 °C at 2 °C/min, held for 4 min, increasing to 214 °C at 4 °C/min, followed by a 1 °C/min gradient up to 217 °C, held for 4 min, and finally to 250 °C at 3 °C/min held for 4 min; the methylation GC-MS program was 140–180 °C at 2 °C/min, followed by a 1 °C/min gradient up to 200 °C, and finally to 250 °C at 3 °C/min held for 5 min.

(a) Extraction, isolation and purification

Green tea leaves (2.5 kg) were extracted with 25 L water at 100 °C for 2 h. The residue was removed by filtration. The spent leaves were re-extracted under the same conditions. The supernatant was combined and concentrated under reduced pressure. The crude TPS extract was obtained through precipitation by adding ethanol to the concentrated solution until the ethanol concentration reached 40% (40% ethanol precipitation fraction, TPS1, 102 g, yield 4.08%) and 70% (70% ethanol precipitation fraction, TPS2, 53.2 g, yield 2.13%), respectively. The precipitates were collected by centrifugation (9829× *g*, 10 min), washed twice with 95% ethanol, and freeze-dried. The flow chart showing the process of isolating various fractions of polysaccharides is presented in Figure 1.

TPS1 (5 g) was dissolved in 40 mL distilled water and centrifuged (43,540× *g*, 10 min). The residue was dissolved in 20 mL distilled water again and centrifuged (43,540× *g*, 10 min) to remove the residue. The supernatant was combined and loaded on a DEAE-cellulose column (50 cm × 5 cm) pre-treated with 0.5 M NaOH, 0.5 M HCl and equilibrated with distilled water. The TPS1 was first eluted with distilled water and then with 0.1, 0.2, 0.4 and 2.0 M of NaCl by stepwise increments. The fraction eluted with 0.2 M NaCl (TPS1-2) was further fractionated on a High-Resolution Sephacryl™ S-300 column (60 cm × 2.6 cm) and eluted with water to give two major fractions of TPS1-2a (180–220 min) and TPS1-2b (240–280 min). 

(b) Homogeneity and molecular weight

Homogeneity and molecular weights of the isolated polysaccharides were determined by high performance gel permeation chromatography (HPGPC), KS-805 and KS-804 column in serials, ID 8 mm, and length 300 mm, (Shodex Co., Tokyo, Japan) [38]. The standard curve was established using different pullulans with known molecular weight (P-5, P-10, P-20, P-50, P-100, P-200, P-400 and P-800, Shodex Co.). The column temperature was kept at 40.0 ± 0.1 °C. NaCl 0.2 M was used as an eluant and the flow rate was kept at 0.8 mL/min. All samples were prepared as 2 mg/mL solutions, and 20 μL aliquot was injected for each run.

(c) Monosaccharide analysis

The polysaccharide sample was hydrolyzed with 2 M TFA at 121 °C for 2 h. After repeated evaporation with methanol to completely remove TFA, the residue was dissolved in 0.1 mL of distilled water and analyzed on a PEI-cellulose plate (Merck, Darmstadt, Germany), developed with EtOAc-pyridine-AcOH-water 5:5:1:3 (*v*/*v*). The plate was visualized by spraying with *O*-phthalic acid reagent and heating at 100 °C for 5 min [39]. The remaining hydrolysate was reduced by NaBH_4_ for 3 h at room temperature. After neutralization with AcOH and evaporation to dryness, the residue was acetylated with Ac_2_O for 1 h at 100 °C. The resulting alditol acetates were subjected to GC-MS analysis. Uronic acid content was determined by the *m*-hydroxydiphenyl method [23].

Reduction of carboxyl groups was carried out using CMC-NaBH_4_ for three times following literature methods [24,40]. The reduced polysaccharides (TPS1-2are and TPS1-2bre) were hydrolyzed and converted into alditol acetates as described above. The configuration of reduced polysaccharides (TPS1-2are and TPS1-2bre) were determined by comparing with d-galactose and l-galactose standards using the double hydrolysis/reductive amination method as described by Cases Cerezo, and Stortz [25].

(d) Determination of *O*-methyl and *O*-acetyl esterification degree of polysaccharides

TPS samples (TPS1-2a and TPS1-2b, 2.0 mg) were saponified by the addition of 0.8 mL of 2 M NaOH at 25 °C, respectively. The reaction was terminated after 1 h by the addition of 0.8 mL of 2 M HCl, and the pH was adjusted to 2.0, as described by Nunes, *et al.* [41]. The GC oven temperature program used for methanol was set at 65 °C (held for 8 min), and for acetic acid at 100 °C (held for 8 min). The flow rate of the carrier gas (N_2_) was set at 2 mL/min.

### 3.3. Periodate Oxidation

The homogeneous TPS samples (TPS1-2a and TPS1-2b, 50 mg) were oxidized with 50 mL 0.02 M sodium periodate at 4 °C in the dark. 0.1 mL solution was taken at 4, 24, 48, 72, 96 and 120 h, diluted to 25 mL with distilled water. The periodate oxidation was monitored by spectrophotometric method at the wavelength λ = 223 nm [42]. Ethylene glycol was added to the solution to end the reaction. Consumption of NaIO_4_ was calculated from the absorption at the same wavelength.

### 3.4. Methylation Analysis

The reduced TPS fractions (TPS1-2are and TPS1-2bre, 5 mg) in which the carboxyl groups had already been reduced to hydroxyl group were dissolved in 3 mL anhydrous DMSO. NaOH powder (500 mg) was added subsequently and the mixture was sealed and stirred for 1 h under nitrogen. The mixture was methylated initially with 0.2 mL methyl iodide for 20 min and then another 0.5 mL methyl iodide was added for further methylation for another 1 h. Water (2 mL) was added to end the reaction. The solution was extracted by 3 mL chloroform, and the chloroform layer was washed 3 times with water and dried. The methylated polysaccharide was retreated twice as described above [43]. The fully methylated polysaccharide was hydrolyzed and converted into partially acetylated partially methylated alditol acetates and analyzed by GC-MS.

### 3.5. NMR Analysis

The native TPS samples (TPS1-2a and TPS1-2b, 30 mg) were dissolved in 0.5 mL D_2_O (99.8 Atom% D, Schweres Wasser, USA). The ^1^H NMR, DEPT-NMR, ^13^C NMR, heteronuclear single quantum coherence (HSQC) and heteronuclear multiple bond correlation (HMBC) spectra were measured using a Bruker Avance III 400 spectrometer (Bruker Instruments, Inc., Billerica, MA, USA) at 25 °C. The chemical shifts of ^13^C and ^1^H NMR are expressed in ppm using acetone as an internal standard; 31.50 ppm for ^13^C NMR and 4.70 ppm for ^1^H NMR (HDO). The DEPT experiments were performed using a polarization-transfer pulse of 135°. All the experiments were recorded and data were processed using standard Bruker software.

### 3.6. Phagocytosis Assay

The human promyelocytic leukaemia cell line HL60 was used to evaluate the phagocytosis-enhancing capacity of TPS and its fractions. The HL-60 cells (5.0 × 10^5^ cells/mL) were differentiated along monocytic lineage by the addition of 1α,25-dihydroxyvitamin D3 (VD3) and incubated in a complete medium for 48 h. After differentiation, 200 μL of HL-60 cells (8 × 10^5^ cells/mL) were transferred to 96-well flat-bottom plates. The non-differentiated HL60 cells were incubated at the same concentration (in the complete medium without VD3) as a background control. 

The cells were treated with the test samples (final concentration at 1.89 and 18.9 μg/mL, respectively) and with 60 μL of a 0.0033% suspension of Yellow Green labelled microspheres added and incubated at 37 °C in 5% CO_2_ for 24 h. To set the basal level, the differentiated HL60 cells in the complete medium were used and 1 μg/mL LPS was used as a positive control. 

After the incubation period, the cells were transferred to a 96-well V-bottom plate and washed three times. For analysis, the cells were transferred to a 96-well clear-bottom black fluorescence plate and fixed with formaldehyde (0.37%, *v*/*v*) for 30 min at room temperature in the dark. The ratio of phagocytosis was measured by using a Beckman Z2 coulter counter (Beckman Coulter, Fullerton, CA, USA). The data were normalized using the positive control and expressed as a relative fluorescence unit (RFU).

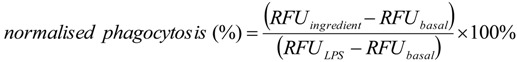
(1)


A normalized percentage >40% was set as positive [14,22].

## 4. Conclusions

In our study, the phagocytic activity of green tea polysaccharides were investigated, and two natural homogalacturonan (HG) pectins (*M*_W_ *ca.* 20 kDa) were isolated from green tea leaves based on their excellent immunomodulatory activity and availability. The primary structures of the HG pectin characterized in this work are different from those reported with immunomodulatory activity in the existing literature. This finding suggests that the HG pectin is a very important molecule to promote phagocytic activity in green tea.
